# Molecular Targets of Cannabidiol in Experimental Models of Neurological Disease

**DOI:** 10.3390/molecules25215186

**Published:** 2020-11-07

**Authors:** Serena Silvestro, Giovanni Schepici, Placido Bramanti, Emanuela Mazzon

**Affiliations:** Istituto di Ricovero e Cura a Carattere Scientifico (IRCCS) Centro Neurolesi “Bonino-Pulejo”, Via Provinciale Palermo, Contrada Casazza, 98124 Messina, Italy; serena.silvestro@irccsme.it (S.S.); giovanni.schepici@irccsme.it (G.S.); placido.bramanti@irccsme.it (P.B.)

**Keywords:** cannabidiol, molecular mechanisms, neurological diseases, neuroprotective effects

## Abstract

Cannabidiol (CBD) is a non-psychoactive phytocannabinoid known for its beneficial effects including antioxidant and anti-inflammatory properties. Moreover, CBD is a compound with antidepressant, anxiolytic, anticonvulsant and antipsychotic effects. Thanks to all these properties, the interest of the scientific community for it has grown. Indeed, CBD is a great candidate for the management of neurological diseases. The purpose of our review is to summarize the in vitro and in vivo studies published in the last 15 years that describe the biochemical and molecular mechanisms underlying the effects of CBD and its therapeutic application in neurological diseases. CBD exerts its neuroprotective effects through three G protein coupled-receptors (adenosine receptor subtype 2A, serotonin receptor subtype 1A and G protein-coupled receptor 55), one ligand-gated ion channel (transient receptor potential vanilloid channel-1) and one nuclear factor (peroxisome proliferator-activated receptor γ). Moreover, the therapeutical properties of CBD are also due to GABAergic modulation. In conclusion, CBD, through multi-target mechanisms, represents a valid therapeutic tool for the management of epilepsy, Alzheimer’s disease, multiple sclerosis and Parkinson’s disease.

## 1. Introduction

Neurological diseases are complex conditions affecting millions of people around the world [1]. These disorders can have different etiologies; indeed, they can be caused by genetic or environmental factors [2,3,4,5]. Although for some of these pathologies treatment that can delay or control the clinical symptoms is available, they remain incurable diseases.

Phytocannabinoids, such as CBD, represent a new class of compounds characterized by beneficial effects in various neurodegenerative and psychiatric diseases [6,7].

CBD is extracted from *Cannabis sativa*, and, together with the psychoactive Δ^9^-tetrahydro-cannabinol (Δ^9^-THC), they represent the main neuroactive components of the plant. Unlike Δ^9^-THC that induces psychotropic effects, CBD is the main non-psychotropic compound present in the plant [8,9].

CBD shows a relatively low toxicity and dependence profile [10]. For these reasons, the role of CBD as adjuvant therapy is being evaluated in those conditions in which the available treatments are not satisfying. Furthermore, CBD has a broad spectrum of therapeutic properties, such as anxiolytic [7,11], neuroprotective [7,12,13,14,15], antidepressant [16], anti-inflammatory [17,18,19] and immunomodulating activities [20,21]. The neuroprotective effects of CBD are due to its antioxidant and anti-inflammatory activities and the modulation of a large number of brain biological targets, such as receptors and channels, involved in the development and maintenance of neurodegenerative diseases [22]. CBD can exert its antioxidant action directly by the modulation of oxidative stress or indirectly through molecules targets associated with the redox system such as nuclear factor erythroid 2-related factor 2 (Nrf2), implicated in the transcription of genes that encodes for antioxidant proteins, such as superoxide dismutase (SOD) and glutathione (GSH) peroxidase [23,24]. CBD can increase the activity of GSH peroxidase and reductase, favoring the reduction of malondialdehyde and thus preventing oxidative stress [25]. Moreover, thanks to its ability to reduce reactive oxygen species (ROS), CBD maintains the correct GSH levels, necessary for the antioxidant activity of vitamins A, C and E [26]. CBD is a regulator of the expression of nitrotyrosine and inducible nitric oxide synthase (iNOS), thus promoting the reduction of the production of ROS [27]. Moreover, CBD exerts its anti-inflammatory action, modulating the release of proinflammatory cytokines such as interleukin-6 (IL-6) and interleukin 1-β (IL-1β) and interacting with transcription factors such as tumor necrosis factor α (TNF-α), nuclear factor κB (NF-κB) or peroxisome proliferator-activated receptor γ (PPARγ) [28,29,30]. CBD also performs its anti-inflammatory action by regulating the transient receptor potential (TRP) channels such as transient receptor potential vanilloid (TRPV) Type 1 and 2, a non-selective cationic channel whose activation allows the entry of Ca^2+^ [31]. Indeed, in the neuroinflammatory conditions, an increase of the density and sensitivity of TRPV1 was demonstrated. Conversely, the binding of the CBD with TRPV1 leads to the desensitization of these channel receptors and a consequent reduction of neuroinflammation, thus explaining the neuroprotective properties of CBD [32].

Therefore, in recent years, the scientific community has shown interest in this compound due to its neuroprotective effects in several neurological disorders, including Parkinson’s disease [33,34], Alzheimer’s diseases [35,36,37] and epilepsy [38]. Additionally, CBD shows other actions such as antidepressant, antipsychotic, antiepileptic and analgesic effects, as highlighted in preclinical studies and human clinical trials [7,39,40,41].

The purpose of this review is to describe the molecular mechanisms associated with the efficacy of CBD in neurological diseases. In the present review, the experimental studies highlighting the CBD’s mechanisms of action in neurological disorders are summarized.

## 2. Methodology

**Figure 1:**
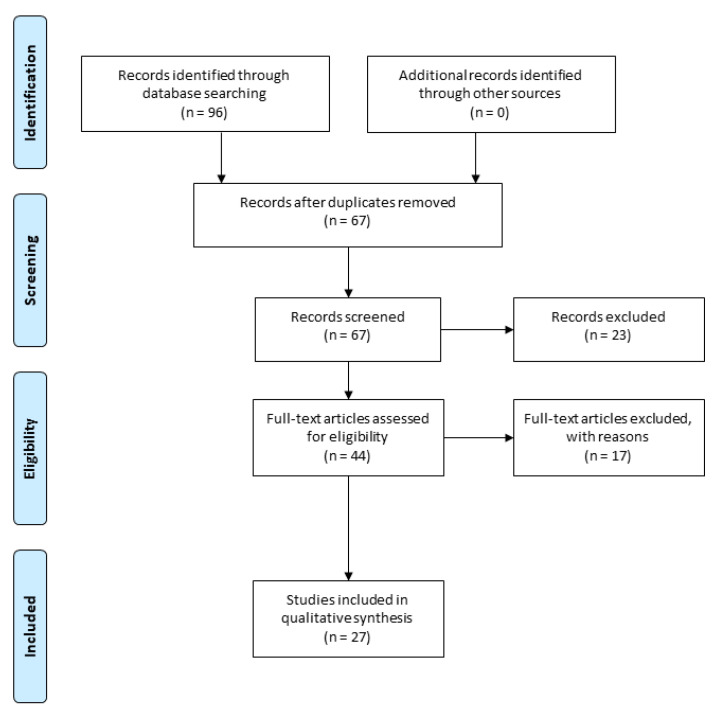
Prisma flow diagram illustrating the selection methodology of the preclinical studies used for the writing of the review. Duplicate articles were excluded from the total of the studies recorded. Instead, articles that evaluate the biochemical and molecular mechanisms underlying the effects of CBD and its therapeutic application in neurological diseases are considered (The PRISMA Statement is published in [42]).

In this review, the articles published from 2004 to 2020 are considered. Specifically, the bibliography research in PubMed was performed using the following keywords: “cannabidiol”, “neurological disease”, “adenosine receptors”, “serotonin receptors”, “transient receptor potential”, “TRPV receptors”, “GPR55 receptors”, “peroxisome proliferator-activated receptor-γ”, “PPARγ receptors” and “GABA receptors”. In this way, 67 articles were found, as shown in the Prisma flow diagram (Figure 1). Articles that evaluate the biochemical and molecular mechanisms underlying the effects of CBD and its therapeutic application in neurological diseases are considered.

## 3. Chemical Properties of Cannabidiol

**Figure 2:**
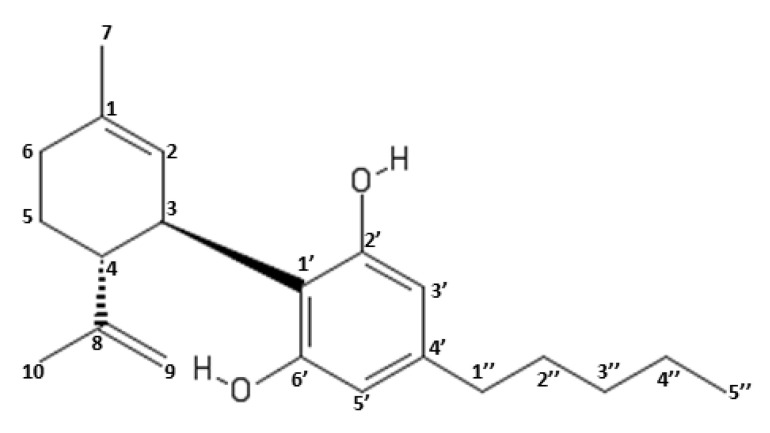
Chemical Structure and numbering system of Cannabidiol (CBD).

CBD was first isolated in 1940 from Mexican marijuana by Roger Adams and from Indian charas by Alexander Todd [43,44]. However, its crystalline structure was determined in 1977 (Jones et al. 1977) [45]. CBD 2-[(1*R*,6*R*)-3-methyl-6-prop-1-en-2-ylcyclohex-2-en-1-yl]-5-pentylbenzene-1,3-diol is a terpenophenol containing 21 carbon atoms, with the formula C_21_H_30_O_2_. CBD is a cyclohexene which is substituted in position 1 by a methyl group, by a 2,6-dihydroxy-4-pentylphenyl group at position 3 and with a prop-1-en-2-yl group at position 4 [46]. The aromatic ring and the terpene ring are almost perpendicular to each other. In the chemical nomenclature, its chemical numbering is determined by the terpene ring (Figure 2).

The reactivity of CBD is mainly due to the methyl group in position C-1 of the cyclohexane ring, to the hydroxyl groups present in the aromatic ring in position C-2’ and C-6’ and to the pentyl chain present in position C-4’. The hydroxyl groups are also capable of binding threonine, tyrosine and glutamic acid [47]. Moreover, CBD, thanks to its hydroxyl groups in the aromatic ring, can exert an antioxidant action by inactivating the free radicals [48]. 

## 4. Cannabidiol Mechanism of Action

Unlike other cannabinoids, CBD has a poor affinity for cannabinoid receptor type 1 (CB1) and cannabinoid receptor type 2 (CB2); however, it acts as a non-competitive negative allosteric modulator of CB1 [49]. It has recently been shown that CBD is a CB1R-negative allosteric modulator of Δ^9^-THC and the 2-arachidonoyl-glycerol (2-AG), this evidence may explain some of the in vivo effects of this nonpsychoactive phycompound [50]. Furthermore, CBD modulates the tone of endocannabinoids by inhibiting cellular uptake of the endocannabinoids arachidonoylethanolamide (AEA) [51]. AEA, CB1, CB2 and 2-AG constitute the endocannabinoid system (ECS) responsible for the synthesis and degradation of endocannabinoids [52]. Therefore, CBD can lead to increased levels of AEA, thus interacting with CB receptors [53]. AEA is involved in different biological processes such as mood regulation, pain sensation and appetite. The synthesis of AEA and 2-AG is regulated by increased of intracellular calcium (Ca^2+^). Moreover, AEA and 2-AG are, respectively, metabolized by fatty acid amide hydrolase (FAAH) and monoglyceride lipase [54]. The increase of intracellular Ca^2+^ induces the production and release into the synaptic space of AEA and 2-AG that acts as retrograde synaptic messengers [55,56]. Indeed, these endocannabinoids acting on CB1 at a presynaptic level block the release by neuronal terminals of neurotransmitters such as γ-aminobutyric acid (GABA), dopamine, glutamate, serotonin (5-HT), norepinephrine and acetylcholine [57,58]. 

However, it was shown that CBD has more affinity for 5-HT receptors, non-endocannabinoid G protein-coupled receptors (GPCRs) and other targets such as enzymes and ion channels [53]. Indeed, some anti-inflammatory and immunosuppressive effects of CBD may be partly mediated by 5-HT and adenosine receptors (ARs) which are not considered part of the ECS. CBD acts as a 5-HT_1A_ agonist, as a partial agonist of 5-HT_2A_ and as a non-competitive antagonist of 5-HT_3A_ [59,60,61]. Furthermore, CBD is capable of activating the ARs [62]. Moreover, CBD acts as a GPR55 antagonist and as an agonist of TRPV1 and TRPV2 [63]. It has already been reported that CBD exerts its anti-inflammatory and neuroprotective effects also due to its ability to activate PPARγ [17,64]. Furthermore, it is a positive allosteric modulator of GABA_A_ receptors, thereby exerting its anticonvulsant, analgesic and anxiolytic properties [65]. However, despite all this evidence, the molecular mechanisms underlying the effects of CBD remain complex.

## 5. Pharmacokinetic Properties of Cannabidiol

The pharmacokinetics and observed effects of CBD are related to the formulation and route of administration [66].

CBD can be administered orally, inhaled and vaporized [67]. CBD administered by inhalation is effectively absorbed into the lungs from the circulating blood, showing similar pharmacokinetics to the intravenous route [68]. Administered by inhalation, CBD reaches peak plasma concentrations in 5–10 min and shows bioavailability of 31%. However, the need for specialized equipment for these routes of administration limits the development of this mode of delivery [69]. On the contrary, oral administration shows a variable pharmacokinetic profile, probably due to the poor solubility of CBD in water [70]. Furthermore, the maximum plasma concentrations are lower than those reached when the drug is administered by inhalation. Indeed, CBD in human showed an oral bioavailability of 6% [71]. The administration of CBD by the oro-mucosal and sublingual route shows a less variable pharmacokinetic profile than the oral administration. Instead, when CBD is administered intravenously, it quickly passes the blood–brain barrier (BBB) to distribute in the brain, adipose tissue and other organs [68]. Moreover, thanks to its liposolubility, CDB forms aggregates which can be released slowly into adipose tissue [72].

CBD is metabolized in the liver by cytochrome P450 enzymes (CYPs) such as CYP2C19 and CYP3A4, CYP1A1, CYP1A2, CYP2C9 and CYP2D6 and is converted to 7-hydroxycannabidiol (7-OH-CBD) [73]. After hydroxylation, CBD is further metabolized in the liver and subsequently excreted, mainly in feces and urine. CBD exhibits a half-life of 18–32 h and a clearance of 57.6–93.6 L/h [69].

CBD is well tolerated and shows a good safety profile at therapeutic dosages. However, some studies have shown that CBD possesses strong inhibitory activity against CYP2C, CYP2D6 and CYP3A isoforms [74].

## 6. Molecular Targets of CBD for Application in Neurodegenerative Diseases

The neuroprotective effects of CBD based on its anti-inflammatory and antioxidant action are directed to the modulation of receptors and channels involved in neurodegenerative diseases [22]. It is known that CBD interacts with many non-endocannabinoid signaling systems such as G protein coupled-receptors, TRPV1 and PPARγ [75]. Additionally, CBD therapeutic potential possibly derives from its GABAergic modulation [65].

### 6.1. GPCRs

#### 6.1.1. Adenosine Receptors 

ARs (A_1_R, A_2A_R, A_2B_R and A_3_R) are GPCRs stimulated by endogenous adenosine, involved in several physiological and pathological processes [76]. The stimulation of A_1_R and A_3_R by adenosine leads to the activation inhibiting G (G_i/o_) proteins with the consequent inhibition of adenylate cyclase and intracellular reduction of cyclic adenosine monophosphate (cAMP). Instead, A_2A_R and A_2B_R promote the G protein activation and the subsequent increase of cAMP [77]. ARs activation involves the modulation of second messengers and additional signaling mechanisms such as phospholipase C; the protein kinase C dependent on Ca^2+^ involved in cell communication; phosphoinositide 3-kinases/protein kinase B (PI_3_K/Akt) signaling involved in cell proliferation, growth and differentiation; and the activation of ion channels and regulation of Ca^2+^ [76,78]. ARs are located in immune cells, blood vessels, astrocytes, microglia, corpus striatum and spinal cord [79]. ARs can affect both in the central nervous system (CNS) and peripheral tissues, thus could represent a useful tool for the development of new neuroprotective strategies [79,80].

Although CBD exerts its beneficial action through several signaling pathways, its anti-inflammatory effects seem to involve A_2A_R [81]. The anti-inflammatory effect of CBD can be directly mediated by A_2A_R whose activation induces the regulation of the immune response, as well as a reduction of proinflammatory cytokines [82,83]. CBD can improve the adenosine signaling, leading to an increase of extracellular adenosine and a consequent reduction of the neuroinflammation [81]. Mecha et al. demonstrated the anti-inflammatory effects of CBD, through A_2A_R activation, in a viral model of multiple sclerosis. They showed in mice infected with Theiler’s murine encephalomyelitis virus (TMEV) that CBD (5 mg/kg), administered via intraperitoneal (i.p.) daily, for seven days, led to a reduction of leukocyte migration in the blood and of the inflammatory response. Moreover, CBD treatment induces the downregulation of the levels of adhesion expression of vascular cells molecule-1 (VCAM-1), chemokine ligand 2 (CCL2) and chemokine ligand 5 (CCL5). Likewise, CBD reduced IL-1β and microglia activation, thus demonstrating its immunosuppressive and neuroprotective action. In addition, CBD has also improved motor deficits, especially in the chronic phase of the disease. To confirm the action of CBD on A_2A_R the animals were treated with ZM241385 (5 mg/kg), a selective A_2A_R antagonist, at the time of TMEV infection and 30 min before CBD treatment. ZM241385, especially in the early stages, attenuated some of the anti-inflammatory effects of CBD, such as the inhibition of the expression of VCAM-1, the infiltration of immune cells and the reduction of immunoreactivity. Moreover, it was also demonstrated that ZM241385 (5 μM) with a dose-dependent mechanism, antagonizes A_2A_R blocking completely the inhibitory action of CBD on the release of VCAM-1. Conversely, the single administration of ZM241385 did not affect TMEV mice. Therefore, the results suggest that the A_2A_R mediated anti-inflammatory effects of CBD could be useful for the management of inflammatory diseases such as multiple sclerosis [18]. 

The involvement of A_2A_R in neuroprotective effects of CBD was also demonstrated in vitro on hypoxic-ischemic immature brain of mice. In this study, Castillo et al. showed the effects of CBD in forebrain sections of C57BL6 mice incubated in absence of oxygen and glucose and treated for 15 min with CBD or vehicle. CBD treatment (100 μM) has significantly reduced acute brain damage and apoptosis, evaluated through the decrease in the efflux of lactate dehydrogenase. In the same way, CBD led to a reduction of glutamate and increase of caspase-9. CBD has also reduced the neuroinflammation leading to the reduction of IL-6, TNF-α, cyclooxygenase-2 (COX-2) and iNOS. Moreover, it was shown that the administration of SCH58261, an A_2A_R antagonist, or AM630, a CB2 antagonist, abolished the neuroprotective effects of CBD. The possible affinity of CBD to CB2 receptors is prompted by the effect of AM630 on CBD neuroprotection. Therefore, the study demonstrated how the neuroprotective effects of CBD can be mediated by A_2A_R and CB2 receptors [28]. The neuroprotective effects of CBD were also demonstrated by Martin-Moreno et al. in vitro and in vivo model of Alzheimer’s disease. CBD inhibited ATP-induced intracellular Ca^2+^ increase in cultured N13 cells and primary microglial cells. The use of ZM241385, an A_2A_ receptor antagonist, reversed the effects of CBD on intracellular Ca^2+^ in N13 microglial cells and primary rat microglial. This result confirms the implication of A_2A_R in the action of CBD. In vivo, CBD at a dose of 20 mg/kg was able to prevent cognitive impairment induced by amyloid-β (Aβ). Indeed, after i.p. administration for three weeks, CBD reduced the expression of the gene encoding for IL-6, a proinflammatory cytokine. Therefore, in light of these results, CBD, interacting with A_2A_R, could be a useful approach for Alzheimer’s disease [84].

Instead, Magen et al. evaluated the A_2A_R-mediated therapeutic effects of CBD in the experimental model of hepatic encephalopathy induced by bile duct ligation. After four weeks of treatment, CBD (5 mg/kg), administrated i.p. daily, induced a reduction of expression of the TNF-α-receptor 1 gene in the hippocampus. Conversely, increased the expression of the brain-derived neurotrophic factor (BDNF) gene. CBD exerts these effects through an indirect modulation of the A_2A_Rs. Indeed, the use of its antagonist ZM241385 (1 mg/kg) reversed the effects of CBD, confirming the involvement of the A_2A_Rs. In this way, the chronic treatment with CBD, through the indirect activation of the A_2A_Rs, improved the cognitive and motor function of the rats with hepatic encephalopathy. As demonstrated by these studies, CBD, probably inhibiting the reuptake of adenosine, exerts an indirect modulation of ARs [82].

In conclusion, the results of these studies show that CBD, through A_2A_R activation, exerts anti-inflammatory effects in models of multiple sclerosis, hypoxic-ischemic damage, Alzheimer’s disease and hepatic encephalopathy (Table 1).

#### 6.1.2. 5-HT Receptors 

5-HT receptors are activated by serotonin and involved in the release of neurotransmitters and hormones, thus regulating many of the processes that occur in the nervous system. The family of receptors 5-HT_1_ is coupled to G_i/0_ proteins and to adenylate cyclase which leads to the production of cAMP. In particular, the 5-HT_1A_ receptor inhibits the Ca^2+^ channel and activates a ligand-dependent potassium (K^+^) channel [85]. 5-HT_1A_ receptors are associated to GPCR and they modulate neurotransmission through K^+^ and Ca^2+^ channels. Moreover, the 5-HT_1_ receptors were classified into five subtypes (5-HT_1A_, 5-HT_1B_, 5-HT_1D,_ 5-HT_1E_ and 5-HT_1F_), which are located in different areas of the brain at pre- and post-synaptic level [86]. It was demonstrated that the main neuroprotective effects of CBD are related to the 5-HT_1A_ receptor. Russo et al. demonstrated that CBD while showing a low-affinity agonism towards the 5-HT_1A_ receptor could enhance 5-HT_1A_-mediated neurotransmission [59]. 

Mishima et al. explored the 5-HT_1A_ receptors-mediate neuroprotective effects of CBD, in mice with middle cerebral artery (MCA) occlusion. Mice received 3 or 10 mg/kg of CBD immediately before and 3 h after occlusion. CBD, at a dose of 3 mg/kg, significantly decreased the infarct volume induced by MCA occlusion. However, treatment with CBD (3 mg/kg) plus WAY100135 (10 mg/kg; a 5-HT_1A_ antagonist) inhibited the effects of CBD. This data suggested the involvement, at least in part, of 5-HT_1A_ receptors in the neuroprotective effects of CBD against cerebral ischemia [87].

Gomes et al. evaluated the beneficial effects of CBD, through the facilitation of the 5-HT1A receptors, in motor-related striatal disorders, such as Parkinson’s disease. For the study, catalepsy was induced in mice, using pharmacological mechanisms, in order to test the motor function disorders. Thirty minutes before receiving the drugs that induce catalepsy, mice were treated with CBD 5, 15, 30 or 60 mg/kg (i.p.). The pretreatment with CBD attenuated the cataleptic effects, in a dose-dependent manner. To explain the mechanism of action by which this phytocannabinoid exerts its anticataleptic action, mice were treated intraperitoneally with WAY100635 (0.1 mg/kg), a 5-HT_1A_ receptor antagonist, 30 min before of the treatment with CBD (30 mg/kg). The administration of WAY100635 prevented the anticataleptic effect of CBD. Therefore, CBD exerting its anticataleptic action, through a mechanism that involves 5-HT_1A_ receptors, could be a possible therapeutic tool in Parkinson’s disease [88]. Moreover, Sonego et al. evaluated the 5-HT_1A_ receptors-mediated anticataleptic effect of CBD. In this study, the researchers induced catalepsy, in male Swiss mice, with haloperidol (0.6 mg/kg). The pre-treatment with CBD (15–60 mg/kg) i.p., prevented the catalepsy. To understand the mechanism of action, it was demonstrated that administration i.p. of WAY100635 (0.1 mg/kg) reduced the anticataleptic effect of CBD and its action on the expression of c-FOS. Moreover, it was shown that the administration of bilateral injections of CBD (60 nmol) into the dorsal striatum, followed by treatment with haloperidol (0.6 mg/kg), reduced the catalepsy, in a similar way to systemic administration. These data suggest that this compound, via activation of 5-HT_1A_ receptor, could represent a therapeutic opportunity for the treatment of striatal disorders such as Parkinson’s disease [89]. 

Instead, Pelz et al. evaluated the role of the 5-HT_1A_ receptors in the anticonvulsant effect of CBD. In this study, to induce the experimental model of generalized seizure, male Wistar Kyoto rats were given a single i.p. injection of 85 mg/kg pentylenetetrazole (PTZ), to induce seizures. Mice received CBD at a dose of 100 mg/kg 60 min before induction of seizures. The results show that CBD significantly reduced the proconvulsant activity induced by PTZ. In particular, CBD significantly reduced seizure severity and the number of animals exhibiting seizure activity and prevented the severe consequences of seizures. Serotonergic signaling is known to be involved in seizure susceptibility and CBD shows a binding affinity for both 5-HT_1A_ and 5-HT_2A_. To test the mechanism of action used by CBD to exert these anticonvulsant effects, mice were pretreated with WAY 100635 (1 mg/kg), a 5-HT_1A_ antagonist, or MDL-100907 (0.3 mg/kg), a 5-HT_2A_ antagonist. Contrary to what might be expected, pretreatment with WAY 100635 and MDL-100907 did not reduce the anticonvulsant effect of CBD. However, this study does not prove that CBD exerted its effects through the 5-HT_1A_ or 5-HT_2A_ receptors [90].

CBD has anxiolytic and analgesic effects and it is known that, at least in part, the anxiolytic effects of CBD depend on the activation of 5-HT_1A_ mediated neurotransmission. De Gregorio et al. evaluated these effects using rats subjected to the spared nerve injury for 24 days to induce a neuropathic pain. The animals were treated acutely with increasing intravenous (i.v.) CBD doses (0.1–1.0 mg/kg). As CBD is poorly soluble in water, it was prepared in a vehicle of ethanol/Tween 80/0.9% saline (3:1:16). Acute CBD treatment reduced the activating activity of 5-HT neurons in the dorsal raphe nucleus. Additionally, to simulate the drug regimen used by patients using CBD to treat chronic neuropathic pain and anxiety, the animals were treated with subcutaneous injections of CBD (5 mg/kg) for seven days. Treatment with CBD for one week decreased mechanical allodynia and anxiety-like behavior, which increased following the spared nerve injury. Furthermore, CBD normalized the activity of 5-HT neurons in the dorsal raphe nucleus. To investigate the mechanism used by CBD, the animals were subjected to single injections of WAY 100635 (0.3 mg/kg; i.v.), 5-HT_1A_ antagonist, capsazepine (1 mg/kg; i.v.), a TRPV1 antagonist and AM 251 (1 mg/kg; i.v.), to CB1 receptor antagonist. Capsazepine treatment completely reduced the antiallodynic effect of CBD, while WAY100635 reduced this effect partially. Instead, the anxiolytic effect of CBD was blocked following treatment with WAY100635. Therefore, it is possible to conclude that treatment with CBD in low doses protects 5-HT neurotransmission, exerts antiallodynic effects through the activation of TRPV1 and anxiolytic properties through the activation of 5-HT_1A_ receptors. In this way, CBD results as a possible candidate for treating neuropathic pain and behavior disorders [91].

Instead, Magen et al. evaluated the 5-HT_1A_ receptors-mediated therapeutic effects of CBD on hepatic encephalopathy induced by bile duct ligation in a model of chronic liver disease. Mice subjected to bile-duct ligation were treated with CBD (5 mg/kg) administrated i.p. every day for 28 days. CBD treatment improved cognitive impairments and motor function. After four weeks of treatment, CBD induced a reduction of expression of the TNF-α-receptor 1 gene in the hippocampus. Conversely, increased the expression of the BDNF gene. To verify other mechanisms of action used by CBD to exert these beneficial effects, the animals were co-treated with WAY 100635. Co-administration of CBD and WAY 100635 reversed the effects of CBD, confirming the involvement of the 5-HT_1A_ receptors [83]. The same positive effects of CBD in the cognitive and locomotor deficits were observed in another model of hepatic encephalopathy induced by injection of thioacetamide. One day after thioacetamide-administration, the animals were given a single dose of 5 mg/kg i.p. Treatment with CBD improved neurological and motor function, assessed, respectively, two and three days after the induction of liver damage. While eight days after the induction of hepatic insufficiency, CBD significantly ameliorated cognitive deficits, impaired following thioacetamide. However, 12 days after treatment with thioacetamide, CBD normalized the 5-HT levels in the brain and induced an improvement in liver function. In this way, the chronic treatment with CBD, through the indirect activation of the 5-HT_1A_ receptors, improved the cognitive and motor function of the rats with hepatic encephalopathy [92].

In conclusion, the findings of these studies suggest that the neuroprotective effects of CBD are mediated, at least in part, by 5-HT_1A_ receptors. In this way, CBD protects against cerebral ischemia and ameliorates the motor-related striatal damage in experimental models. In the same way, it improved the cognitive and motor function in an in vivo model of hepatic encephalopathy. Moreover, CBD exhibited anxiolytic properties through the activation of 5-HT_1A_ receptors, in experimental models of anxiety-like behavior disorders (Table 2).

#### 6.1.3. GPR55 

The CBD also exhibits a high affinity towards the G-protein-coupled receptor 55 (GPR55), a class of receptors implicated in the synaptic transmission. GPR55 is a G protein-coupled receptor widely expressed in the immune and nervous systems. GPR55 is involved in the modulation of parameters and cell processes such as blood pressure and bone density modulation, cell migration and proliferation, inflammation, neuropathic pain, energy balance and antiepileptic action [93]. GPR55 is a seven-transmembrane receptor that acts as a G protein inducing the intracellular increase of Ca^2+^ and the phosphorylation of the extracellular receptor-activated kinases (ERK) protein, which in turn is involved in proliferation, differentiation and cytoskeletal modulation [93]. GPR55 was found at the post-synaptic level in endothelial cells and at the pre-synaptic level in the hippocampus where it appears to increase the release of vesicular glutamate [94,95]. The facilitating effect of GPR55 contrasts the action of CB1 receptors, inhibiting neurotransmitter release. On the contrary, CBD can suppress GPR55 activation, thus increasing the release of neurotransmitters [96]. This mechanism, at least in part, elucidates the anti-convulsive effect of cannabinoids towards epileptic pharmaco-resistant patients [97]. In this regard, Kaplan et al. evaluated the GPR55-mediated antiepileptic properties of CBD in a mouse model of genetically-induced Dravet syndrome (DS). CBD (100 mg/kg or 200 mg/kg) was administered intraperitoneally, twice daily for one week. The acute treatment of CBD, in a manner dependent on its concentration, reduced the thermally-induced seizures and significantly decreased the rate of spontaneous seizures. Additionally, CBD treatment ameliorated hyperactivity due to disease. Moreover, the mouse model of genetically-induced DS showed a decrease in the GABA inhibitory transmission. The CBD treatment restored the excitability of neurons inhibitors in the dentate gyrus of the hippocampus, which represents an important zone for convulsions propagation. To confirm the involvement of GPR55 in anticonvulsant effects of CBD, mice were treated with a CID16020046 (10 µM), a GPR55 antagonist. The treatment with CID16020046 abolished the beneficial effects of CBD in inhibitory. Thus, this result suggests that the therapeutic effects of CBD are mediated through GPR55. Therefore, GPR55 could be an important therapeutic target for the treatment of epilepsy [98]. 

GPR55 can be involved in motor function. Celorrio et al. studied the effects of CBD and abnormal-CBD on the modulation of GPR55, in an experimental model of Parkinson’s disease. To induce Parkinson’s model, adult male C57BL/6 mice were treated, for five weeks, with 1-methyl-4-phenyl-1,2,3,6-tetrahydropyridine (MPTP) (20 mg/kg) and probenecid (250 mg/kg), a drug capable of reducing the renal disposal capacity of MPTP and its metabolites. MPTP mice were treated with CBD (5 mg/kg) and abnormal-CBD (5 mg/kg), administered chronically for five weeks. Abnormal-CBD is a synthetic CBD isomer that owns a high affinity towards GPR55. The study demonstrated that abnormal-CBD prevented the motor deficits MPTP-induced, while CBD did not produce a significant effect in motor behavior tests. However, both CBD and abnormal-CBD induced morphological changes in the microglia, probably due to an anti-inflammatory response. The anti-parkinsonian effect of abnormal-CBD was also confirmed in cataleptic mice induced by i.p. injection of haloperidol (1 mg/kg). Instead, the administration of CBD (5 mg/kg) did not show anti-cataleptic effects but rather abolished the action of abnormal-CBD. To confirm the involvement of GPR55 in the results obtained, it was also shown that the treatment with PSB1216 (10 mg/kg), a GPR55 antagonist, abolished the effect of abnormal-CBD. Conversely, the treatment with GPR55 agonists such as CID1792197 and CID2440433, similarly to abnormal-CBD, showed anti-cataleptic effects. Therefore, the study demonstrated that compounds able to activate GPR55 could be beneficial in combating PD [99]. 

The anti-inflammatory effects of CBD mediated by GPR55 were also tested by González-García et al. in experimental autoimmune encephalomyelitis (EAE) mice, a model of multiple sclerosis. The experimental model was induced in female C57BL/6J mice through i.p. injection of encephalitogenic cells cultured with Myelin Oligodendrocyte Glycoprotein peptide 35–55 (25 µg/mL) and interleukin-12 (25 ng). After the induction of the model, animals were treated with CBD (5–10 mg/kg) or CBD (50 mg/kg) i.p. The study showed an improvement in the disease already at low doses of CBD (5 mg/kg). In addition, it was shown an improvement of the disease with no signs of toxicity also at the high doses of CBD (50 mg/kg). EAE induction caused a significant reduction in the levels of CB1 and the levels were not restored by CBD treatment. On the contrary, CB2 was found in scarce levels in healthy control mice, whereas its expression was significantly increased in the EAE mice. CBD induced a significant reduction of CB2 expression in the treated animals. In the same way, the high level of EAE-induced GPR55 was reduced after CBD treatment. Although the role of GPR55 in EAE is not fully understood, its function for the disease can be very important. Indeed, mice with genetic deletion of GPR55 showed a less severe form of EAE. Therefore, the action of CBD as an antagonist of the GPR55 receptor could be useful to counteract the disease [100].

In conclusion, via the antagonist action of the GPR55 receptor, CBD carries out its anti-inflammatory effects in experimental models of DS, Parkinson’s disease and EAE disease (Table 3).

### 6.2. TRP

TRP is a family of ion channels mainly located on the plasma membrane of many animal cells. CBD could interact with TRP, thus modulating the inflammation [101]. Interestingly, CBD is a potent and selective agonist of TRPV1, a TRP channel from the vanilloid subfamily. TRPV1 is a non-selective cationic channel present on sensory tissues such as skin, lungs, heart and blood vessels. TRPV1 activation induces the release of neuropeptides involved in pain perception, neuroinflammation, and regulation of body temperature [102]. Indeed, this receptor coupled to G proteins is characterized by six transmembrane domains which can be activated by capsaicin and stimuli such as a low pH, heat (>43 °C) and phytocannabinoids [101]. TRPV1 receptors are mainly expressed in the ganglia of dorsal roots in the spinal cord, while in the CNS they located in the hypothalamus and hippocampus [103]. It is known that TRPV1 antagonists possess analgesic properties. However, the activation of TRPV1 receptors by some agonists, such as capsaicin, leads to the entry of Ca^2+^ and Na^+^ with consequent desensitization of the channel [104]. Consequently, the increase of Ca^2+^ induced by capsaicin actives the calcineurin protein which dephosphorylates TRPV1 and other proteins in the voltage-gated Ca^2+^ channels also involved in the nociceptive transmission. Thus, the receptor desensitization in response to capsaicin can render the TRPV1 channel insensitive to further painful stimuli [105]. In the same way, CBD agonist action towards TRPV1 induces the desensitization of these channels [106,107]. Therefore, at least in part, the anti-nociceptive and antihyperalgesic actions of CBD appear to be mediated by activation, dephosphorization and strong desensitization of TRPV1 channels [108].

Costa et al. showed the effects of CBD and the mechanisms associated with antihyperalgesic action in a mouse model of acute inflammation. CBD (10 mg/kg) administered orally 2 h after the induction of the model abolished the thermal hyperalgesia induced by carrageenan (0.1 mL). The CBD co-administration with capsazepine (2 mg/kg), a synthetic capsaicin antagonist, suppressed the carrageenan-induced hyperalgesia. Capsazepine at the higher dose (10 mg/kg) inhibited the CBD-induced antihyperalgesic action. These data suggest how CBD can exert its antihyperalgesic effect by directly involving TRPV1. Therefore, CBD could represent a valid therapeutical tool in the treatment of pathological conditions such as neuropathy [31].

Moreover, CBD is also able to activate TRPV2, transient receptor potential ankyrin 1 (TRPA1) and antagonize also the transient receptor potential cation channel subfamily M member 8 (TRPM8) [101]. CBD through TRP channels, involved in the proliferation and release of proinflammatory cytokines, can regulate Ca^2+^ in immune and inflammatory cells [24].

The effects of CBD and its affinity for the TRPV2 receptors were also demonstrated by Luo et al. in human brain endothelial cells forming the BBB. CBD promoted a long-lasting increase of intracellular Ca^2+^ level, especially at 15 μM dose. After 24 h of incubation, CBD treatment (0.1, 0.3, 1, 3 and 10 μM), in a dose-dependent manner, induced the cell growth of hCMEC/D3 cells, and, after 4 h, it significantly enhanced cell migration. Instead, after 7 or 24 h, CBD significantly increased the tubulogenesis of hCMEC/D3 cells. Additionally, after 72 h to seeding, treatment with 1 μM CBD increased trans-endothelial resistance in human Primary Brain Microvascular Endothelial Cells (hPBMECs) Monolayers. To demonstrate the possible involvement of TRPV2 in CBD-induced effect, cells were pretreated, 5 min before added CBD, with ruthenium red, a nonspecific TRP antagonist, or with tranilast, a selective TRPV2 inhibitor. Ruthenium red and tranilast suppressed the CBD-induced long-lasting increase of intracellular Ca^2+^ levels. The same results were also obtained in silencing cells with TRPV2 siRNA. These data highlight the role of TRPV2 in the CBD mechanism of action. Therefore, CBD, due to the high affinity of TRPV2, could be a potential pharmacological tool to regulate the BBB characteristic [109].

Nabissi et al. evaluated the role of CBD to contrast the proliferation of glioblastoma, through activation of TRPV2. In this study, the authors showed that CBD (10 µM) improved the action of cytotoxic agents able to contrast the proliferation of glioblastoma. CBD, through activation of TRPV2 and the consequent entry of Ca^2+^, improved the action of chemotherapy drugs. To confirm the agonize effect of CBD on TRPV2, it was performed deletion of the TRPV2 poredomain. Since the poredomain of the TRP channels is important for Ca^2+^ entry, the authors demonstrated that the deletion of this region prevents CBD-induced influx of Ca^2+^ by reducing drug absorption and cytotoxic effects. Therefore, with this result, it has been shown that CBD co-administered together with chemotherapeutic agents, activating TRPV2, enhances drug absorption and cytotoxic activity in human glioma cells. Consequently, the administration of chemotherapy drugs together with CBD could improve the efficacy of therapy useful to counteracting the glioma cells [110]. 

Moreover, CBD, through the activation of the TRPV, is able to promote PI_3_K/Akt signaling, which in turn inhibits the glycogen synthase kinase 3β (GSK-3β). The GSK-3β inhibition induces an increase of the Wnt/β-catenin pathway, thus exerting a neuroprotective action against the oxidative stress and neurotoxicity induces of Aβ in the Alzheimer’s disease [54]. In line with this evidence in an in vitro model of Alzheimer’s disease, CBD treatment suppressed the hyperphosphorylation of tau protein-mediated to β-catenin and GSK-3β, in Aβ-stimulated PC12 neuronal cells [111]. Moreover, CBD decreased Aβ levels in SH-SY5Y cells transfected with the amyloid precursor protein (SH-SY5Y^APP+^) [64], and, in an Alzheimer’s disease mouse model, CBD administration ameliorated cognitive impairment [112]. In this context, our research group, in a previous study, investigated the involvement of TRPV2 in the molecular CBD’s mechanism of action, comparing the expression profiles of human gingival mesenchymal stem cells treated with CBD (5 μM) to those without treatment. The results of the transcriptomic analysis show that CBD decreased the expression of genes related to Alzheimer’s disease. Conversely, CBD upregulated genes coding for the PI_3_K subunits and for AKT1. Thus, CBD, through modulation of PI_3_K/Akt signaling, is capable of regulating GSK3β activity and consequently improve hallmarks of Alzheimer’s disease. To study how CBD modulated the PI_3_K/Akt signaling, human gingival mesenchymal stem cells were treated with antagonists for CB1R (SR141716A), CB2R (AM630), or TRPV1 (capsazepine) receptors. Noteworthy, only the pretreatment with capsazepine reversed the CBD-mediated effects. Therefore, CBD, by TRPV1 activation, promoted the PI_3_K/Akt pathway that inactivates GSK3β. Thus, CBD could reduce Alzheimer’s hallmarks [37].

The results of the studies show that CBD activates and rapidly desensitize TRPV1, inducing antihyperalgesic effects. Moreover, TRPV1 activation induced the PI_3_K/Akt pathway signaling, which can reduce Alzheimer’s hallmarks. Instead, CBD, through activation of TRPV2, enhanced cell proliferation and improved the action of chemotherapy drugs (Table 4).

### 6.3. PPARγ Receptors

PPARγ represents a member of the nuclear receptor family and is also a transcription factor modulated by a ligand that regulates the expression levels of genes involved in inflammation, trophic factors production, redox equilibrium, metabolism of glucose and lipids [24,113,114]. PPARγ is also a ubiquitin E3 ligase consisting of several residues of lysine, including Lysine48 capable of generating polyubiquitin chains [115]. The polyubiquitin linked to Lysine48 of PPARγ is responsible for proteasomal degradation of p65, which in turn leads to the inhibition of the inflammatory pathway mediated by NF-κB. Indeed, the activation of PPARγ inhibits the transcription of proinflammatory genes, cytokines such as TNF-α, IL-1β and IL-6, thus preventing the NF-κB signaling pathway [35]. Therefore, PPARγ agonists such as CBD, inhibiting the transcription of downstream genes mediated by NF-κB can perform an anti-inflammatory action through a molecular mechanism regulated by GSK3β [116]. 

This mechanism of action was evaluated by Scuderi et al. in SH-SY5Y^APP+^ cells, an in vitro model of Alzheimer’s disease. SH-SY5Y^APP+^ cells were treated with CBD (10^−9^–10^−6^ M) for 24 h. Treatment with CBD reduced the expression of the APP protein, as well as its ubiquitination, thus leading to the reduction of Aβ and neuronal apoptosis. To demonstrate the selective involvement of PPARγ in mediating CBD activity in SH-SY5Y^APP+^ cells, CBD was co-administered with MK886 (3 μM) or GW9662 (9 nM), selective antagonists of PPARα and PPARγ, respectively. The results show that treatment with GW9662 (9 nM) led to the almost complete lack of efficacy of CBD, thus confirming that, the neuroprotective role of this phytocannabinoid, involved the PPARγ receptors [64]. The neuroprotective effect of CBD through the activation of PPARγ was observed in an experimental study of Alzheimer’s disease. Esposito et al. showed both in vitro and in vivo the properties of PPARγ agonists and non-agonists on neurotoxicity induced by Aβ. Cultures primary of rat astrocytes were induced with Aβ (1 µg/mL). The treatment with CBD (10^−9^–10^−7^ M), in a concentration-dependent manner, reduced the effect of Aβ mediated through the inhibition of NF-κB. On the contrary, the treatment with GW9662 (9 nM), a PPARγ antagonist, was shown to reverse the anti-inflammatory effect of CBD. To confirm the results obtained with CBD in reactive gliosis, Esposito et al. performed the study in vivo. Male Sprague-Dawley rats were induced with Aβ (10 µg/mL). After the induction of the Alzheimer’s disease model, the animals were treated for 15 days with CBD (10 mg/kg; i.p.). The data obtained show that CBD preserved from the neuronal damage induced by Aβ and also led to a reduction of gliosis and glial fibrillary acidic protein. Conversely, the administration of GW9662 (10 mg/kg), the antagonist PPARγ, has completely reversed the neuroprotective effects of CBD. Therefore, the results obtained both in vitro and in vivo confirm the important role of PPARγ in mediating the neuroprotective actions of CBD in experimental models of Alzheimer’s disease [17]. The role of PPARγ in neuroprotective effects of CBD was also demonstrated by Hughes et al. The hippocampal slices of C57/black 6 mice induced with soluble oligomeric Aβ_1–42_ were treated with CBD 30 min before to the addition of Aβ_1–42_. The treatment with CBD improved the synaptic transmission and the potentiation long-term in the hippocampus, thereby preserving it from cognitive deficits induced by Aβ_1–42_. To understand the mechanism of action of CBD, WAY 100635, (300 nM; 5-HT_1A_ antagonists), ZM241385 (100 nM; A_2A_R antagonist), AM 251 (2 µM; CB1 inverse agonist) or GW9662 (2 µM; PPARγ antagonist) was added to the perfusate 30 min before of CBD. It was shown that only the treatment with GW9662 attenuated the neuroprotective effects of CBD. In addition, this study suggested that CBD, at least in part, through interaction with PPARγ, could be a therapeutic potential for the treatment of Alzheimer’s disease [117].

The anti-inflammatory effect of CBD was also shown in tardive dyskinesia, a disease characterized by chronic use of drugs capable of reducing or blocking the dopaminergic neurotransmission, which in turn induces the abnormal and repetitive involuntary movements that mainly involve the orofacial region [118]. In this context, Sonego et al. investigated the PPARγ-mediated protective effect of CBD in vivo and in vitro models of dyskinesia haloperidol-induced. Swiss mice were received two daily i.p. injections of CBD (60 mg/kg) for 21 days and 30 min later were treated with haloperidol (2 and 3 mg/kg). The behavioral analysis showed that CBD treatment prevented dyskinesia induced by haloperidol and reduced the oxidative stress and activation of microglial and inflammatory cytokine (IL-1β and TNF-α) in the corpus striatum. Moreover, it was shown to increase the expression of peroxisome proliferator-activated receptor-γ coactivator 1-α, a co-activator of PPARγ, thereby confirming PPARγ as a molecular target of CBD. To confirm the involvement of PPARγ in the effect of CBD, animals received the injection of GW9662 (2 mg/kg), 30 min after CBD. The administration of GW9662, inhibited the positive effect of CBD on dyskinesia. Moreover, to confirm the involvement of PPARγ, the authors also performed an in vitro study. Primary microglial cells were pretreated with GW9662 (0.1, 1 and 10 µM), 30 min after treatment with CBD (10 µM) and 4 h after the cells were stimulated with lipopolysaccharide (10 ng/mL). The in vitro study confirmed the results obtained in vivo, showing the involvement of PPARγ in the neuroprotective effects of CBD [119]. 

Instead, Dos-Santos-Pereira et. evaluated the PPARγ-mediated effects of CBD on l-3,4-dihydroxyphenylalanine (l-DOPA)-induced dyskinesia, a mouse model of Parkinson’s disease. Male C57⁄BL6 mice were treated with 6-hydroxydopamine (6-OHDA) neurotoxin, which induces hallmarks of Parkinson’s disease. To induce dyskinesia, 6-OHDA-lesioned animals were treated with l-DOPA for 21 days. Subsequently, 15 min before l-DOPA administration, mice received CBD (15, 30 and 60 mg/kg) i.p. for three days. CBD alone was not able to prevent the l-DOPA-induced dyskinesia. To understand the CBD’s mechanism of action, it was co-administrated with capsazepine (1 or 5 mg/kg), a TRPV-1 antagonist, or with arachidonoyl-serotonin (AA-5-HT; 5 mg/kg), an enzyme responsible for inhibiting anandamide metabolism FAAH and TRPV-1. Co-administration with CBD and capsazepine, through an increase in AEA and an antagonism of TRPV1 receptors, leads to the reduction of dyskinesia. Additionally, co-treatment of CBD with AM 251 (1 mg/kg), a CB1 antagonist, or with GW9662 (4 mg/kg), an antagonist of PPARγ receptors, reversed the anti-dyskinetic effect of CBD and capsazepine. In conclusion, CBD together with capsazepine, through the interaction with CB1 and PPARγ receptors, could be a valid therapeutic strategy to prevent the l-DOPA-induced dyskinesia in patients with Parkinson’s disease [120]. Hind et al. also demonstrated the role of PPARγ in mediating the neuroprotective effects of CBD in experimental models of ischemic damage. To induce ischemic damage, the cells were simulated using oxygen–glucose deprivation. CBD treatment (100 nM, 1 µM and 10 μM) was administered either before or immediately after the induction of ischemic damage. CBD (10 μM) reduced the increase of BBB permeability following the ischemic damage. To assess the CBD’s mechanism of action, cells were treated with AM 251 (100 nM; CB1 receptors antagonists), AM630 (100 nM; CB2 receptors antagonist), capsazepine (1 μM; TRPV1 channels antagonists), GW9662 (100 nM; PPARγ antagonist), SCH58261 (100 nM; A_2A_Rs antagonist) and WAY100135 (300 nM; 5-HT_1A_ receptors antagonist). The neuroprotective effect of CBD was abolished by the administration of GW9662 (100 nM) and partially reduced by WAY100135 (300 nM). To confirm the involvement of PPARγ and 5-HT_1A_ receptors underlying the neuroprotective effect of CBD, cells were also treated with pioglitazone (a PPARγ agonist) and 8-OH-DPAT (an agonist of 5-HT_1A_ receptors). The treatment with these receptors showed similar effects of CBD on the permeability induced by ischemic damage. Therefore, the study reported useful results on the neuroprotective effect of CBD in the permeability of the BBB, through the activation of PPARγ and 5-HT_1A_ [121].

In addition, in an experimental model of multiple sclerosis, PPARγ exerted an important role in mediating the effects of CBD. Giacoppo et al. showed the effects of CBD in C57BL/6 mice with EAE immunized with Myelin Oligodendrocyte Glycoprotein peptide 35–55 (300 μg). Fourteen days after the induction of the EAE model, the mice were treated daily with CBD (10 mg/kg) i.p. The results of the treatment demonstrate that CBD restored the PI_3_K/Akt/mTOR pathway, which was downregulated after EAE induction. CBD treatment has also led to the reduction of inflammatory cytokines interferon-γ (IFN-γ) and interleukin 17 (IL-17) and significantly increased the levels of PPARγ. These results suggest that, at least in part, the effects of CBD could be related to the increased level of PPARγ. In conclusion, CBD, through both enhanced PPARγ and modulation of the PI_3_K/Akt/mTOR pathway, could be an interesting therapeutic target for multiple sclerosis [122].

In conclusion, the data obtained from these studies underline the implication of PPARγ in the anti-inflammatory effects of CBD, in experimental models of Alzheimer’s disease, Ischemic stroke, Parkinson’s disease and EAE disease (Table 5).

### 6.4. GABA Receptors

GABA is the principal inhibitory neurotransmitter of the CNS. This receptor binds three classes of type A receptor: GABA_A_, GABA_B_ and GABA_C_. Among these, the GABA_A_ receptor subfamily appears to be involved in many neurological diseases [123,124,125]. Deficits of this receptor are responsible for several neurological disorders such as Huntington’s disease [126], cognitive alterations [3], epileptic disorders [127], drug addiction [128], chronic stress and anxiety [129]. For these reasons, GABA_A_ receptor represents an interesting target for new compounds. CBD is known to potentiate GABA_A_-mediated inhibitory currents by acting on the GABA_A_ receptor [65]. In accordance with this evidence, our research team in a previous work suggested that CBD might be able to decrease neuronal excitability in NSC-34 motor neuron-like cells through enhancing the expression of genes linked in GABA release and increasing the GABA_A_ receptor gene expression [130].

Recently, CBD has been shown to be a promising compound in the treatment of patients with drug-resistant DS [131,132,133]. In this context, Ruffolo et al. studied the effects of CBD on GABA_A_ receptor-mediated neuronal transmission using human cortical tissue obtained from patients with DS and tuberous sclerosis complex (TSC). Cell membranes obtained from cortical tissue of patients were transplanted in *Xenopus oocytes* to perform experiments. To evaluate the effect of CBD on GABA currents, the cells were preincubated for 10 s with CBD (5 μM) before the co-application of GABA and CBD. CBD enhanced the amplitude of the GABA-evoked current, in cortical tissue of patients with DS. A similar effect was also obtained using cortical tissues of patients with TSC. These results highlight that CBD, increasing the average amplitude of the currents evoked from GABA, could be a new therapeutic discovery in drug-resistant DS [134]. In light of this evidence, in 2018, the US Food and Drug Administration, approved CBD to treat DS and Lennox–Gastaut syndrome, two drug-resistant epileptic syndromes [135].

In the treatment of these drug-resistant epileptic syndromes, CBD is often co-administered with common antiepileptic drugs, such as clobazam (CLB). However, several clinical studies have highlighted the existence of drug–drug interaction between CBD and CLB. In these studies, it was observed that CBD causes an increase in plasma concentrations of both CLB and its active metabolite, *N*-desmethylclobazam (N-CLB) [136,137,138]. Similar to CBD, CLB and N-CLB are positive allosteric modulators of GABA_A_ receptors [139,140,141]. In view of these this data, Anderson et al., in vivo and in vitro, explored the pharmacodynamic and pharmacokinetic interactions of these compounds highlighted the involvement of GABA_A_ receptors. For the study, the researchers used mice with heterozygous loss of function *SCN1A* (*Scn1a^+/−^*), a genetic model of DS. CBD was administrated i.p. 45 min before to receive CLB (0.1–10 mg/kg). The combined treatment of CBD and CLB resulted in an increase of anticonvulsant effect against seizures compared to the administration of the single compound. In this regard, the researchers investigated the novel pharmacodynamic mechanism where CBD and CLB together enhanced inhibitory GABA_A_ receptor activation using *Xenopus oocytes* expressing GABA_A_ receptors. For the in vitro study, GABA treatment (15 µM) was performed three times with a washout period of 7–12 min between GABA applications. Subsequently, CBD (10 µM) was co-applied with GABA, for 60 s. The results show that CBD and CLB exert their anticonvulsant action by enhancing the activity of the GABA_A_ receptor. This pharmacological interaction between CBD and GABAergic drugs, like CLB, could explain the anti-seizures effect of CBD [142].

In another study, Aso et al. showed that chronic co-administration of Δ^9^-THC and CBD improves cognitive deficits in transgenic *AβPP/PS1* mice, a model of Alzheimer’s disease. Interestingly, the positive effects of co-administration of these two compounds may be related to a reduction in glutamate ionotropic receptors AMPA Type Subunits 2/3 and an increase in GABA_A_ receptors. Therefore, this study further confirms the involvement of cannabinoids in excitatory and inhibitory neural activity [143]. These studies demonstrated that CBD, enhancing inhibitory GABA_A_ receptor activation, could be an interesting approach for conditions such as epilepsy (Table 6). 

## 7. Conclusions

The neuroprotective properties of CBD are performed via several mechanisms of action. CBD mainly exerts these effects through multiple biological targets. Specifically, CBD, through A_2A_R activation, exerts anti-inflammatory effects in animal models of Alzheimer’s disease and multiple sclerosis. Moreover, at least in part, it exerts neuroprotective effects by the activation of 5-HT_1A_ receptors. In experimental models of Parkinson’s disease and DS, CBD performs its beneficial effects antagonizing the GPR55 receptor. Noteworthy, CBD through the TRPV1 activation could modulate PI_3_K/Akt signaling, thus ameliorating the Alzheimer’s hallmarks. Additionally, the neuroprotective properties of CBD in Alzheimer’s and Parkinson’s diseases are also mediated by the interaction with PPARγ. Instead, CBD, enhancing the inhibitor GABA transmission, could be an interesting tool to treat epilepsy condition. The preclinical evidence reviewed here, linked with the already reported safety profile of CBD in humans, highlights that CBD represents a new opportunity for the treatment of several neurological diseases. However, further studies are needed to elucidate the molecular mechanisms underlying the properties of CBD and identify new molecular targets.

## Figures and Tables

**Table 1:** Neuroprotective effects of CBD in different neurological diseases through the activation of the A_2A_Rs.

In Vitro and in Vivo Models	CBD Dose	Treatments	Biological/Pharmacological Effect	Neurological Diseases	Ref.
Female SJL/J mice	5 mg/kg	Once-daily during Days 1–7 post-infection	CBD attenuated the activation of microglia downregulating the expression of VCAM-1, CCL2 and CCL5 and the proinflammatory cytokine IL-1β. Moreover, CBD improved motor deficits in the chronic phase of the disease	multiple sclerosis	[18]
Newborn C57BL6 mice	0.1–1000 µM	15 min. pre-incubation	CBD reduced acute brain damage and apoptosis. Moreover, it induced a reduction concentration of glutamate and IL-6 and decreased the expression of TNF-α, COX-2 and iNOS.	hypoxic-ischemic brain damage	[28]
Primary Rat Microglial and N13 Microglial Cells and C57Bl/6 mice	20 mg/kg	Once-daily during the first week, then 3 days/week for 2 weeks	CBD inhibited ATP-induced intracellular Ca^2 +^ increase in cultured N13 and primary microglial cells and A_2A_ receptors may be involved in this mechanism. In vivo, CBD reduced the gene expression of proinflammatory cytokine IL-6 and prevented cognitive impairment induced by Aβ.	Alzheimer’s disease	[84]
Female Sabra mice	5 mg/kg	Every day for 4 weeks	CBD reduced the expression of the TNF-α-receptor 1 gene in the hippocampus. Conversely, enhanced the expression of the BDNF gene. Moreover, CBD, through the indirect activation of the A_2A_R, improved the cognitive and motor function of the rats with Hepatic Encephalopathy.	hepatic encephalopathy	[82]

CBD, cannabidiol; VCAM-1, vascular cell adhesion molecule-1; CCL-2, chemokine ligand 2; CCL-5, chemokine ligand 5; IL-6, interleukin-6; TNF-α, tumor necrosis factor α; COX-2, cyclooxygenase-2; iNOS, inducible nitric oxide synthase; A_2A_R, adenosine 2A receptors; CB2, cannabinoid receptors type 2; Aβ, β-amyloid; BDNF, brain-derived neurotrophic factor.

**Table 2:** Neuroprotective effects of CBD in different neurological diseases through the activation of the 5-HT_1A_.

In Vivo Models	CBD Dose	Treatments	Biological/Pharmacological Effect	Neurological Diseases	Ref.
MCA occlusion male mice	3 or 10 mg/kg	Before and 3 h after damage	CBD, at dose of 3 mg/kg, significantly reduced the infarct volume induced by MCA occlusion, at least in part, through the 5-HT_1A_ receptor.	cerebral ischemia	[87]
Male Swiss mice	5, 15, 30 or 60 mg/kg	30 min before receiving the drugs that induce catalepsy	Pretreatment with CBD reduced the cataleptic effects, in a dose-dependent manner, through the 5-HT_1A_ receptor.	striatal disorders	[88]
Male Swiss mice	15–60 mg/kg or 60 nmol	30 min before or 2.5 h after receiving the drugs that induce catalepsy	Pretreatment with CBD reduced the cataleptic effects, in a dose-dependent manner, through the 5-HT_1A_ receptor.	striatal disorders	[89]
Male Wistar Kyoto rats	100 mg/kg	60 min before induction of seizures	CBD significantly mitigated PTZ-induced seizure.	seizure disorders	[90]
Adult male Wistar rats	0.1–1.0 mg/kg and 5 mg/kg	Acute treatment with cumulative injections of CBD every 5 min and repeated treatment with 5 mg/kg/day for 7 days	CBD (5 mg/kg) protects nerve injury-induced deficits in dorsal raphe nucleus 5-HT neuronal activity. Moreover, CBD exerts antiallodynic effects through the activation of TRPV1 and anxiolytic properties through the activation of 5-HT_1A_ receptors.	allodynia and anxiety-like behavior	[91]
Female Sabra mice	5 mg/kg	28 days	CBD, through the 5-HT_1A_ receptor activation, improved cognition and motor function, which were impaired by bile-duct ligation. Moreover, in the animal model of hepatic encephalopathy, CBD also reduced neuroinflammation, increasing expression of the BDNF genes and reducing TNF-α receptor 1 gene expression.	hepatic encephalopathy	[83]
Female Sabra mice	5 mg/kg	Single dose	CBD ameliorated cognitive impairments and locomotor activity. Moreover, CBD restored the 5-HT levels in the brain and improved the liver function.	hepatic encephalopathy	[92]

CBD, cannabidiol; MCA, middle cerebral artery; 5-HT_1A_, serotonin 5-hydroxytriptamine1A; PTZ, pentylenetetrazole; BDNF, brain-derived neurotrophic factor; TNF-α, tumor necrosis factor-α.

**Table 3:** Neuroprotective effects of CBD in different neurological diseases through the antagonize activation of the GPR55.

In Vitro and in Vivo Models	CBD Dose	Treatments	Biological/Pharmacological Effect	Neurological Diseases	Ref.
*Scn1a* mutant mice	10, 20, 100 or 200 mg/kg	Twice daily for 1 week	Acute treatment of CBD decreased thermally-induced seizures and reduced the rate of spontaneous seizures. Moreover, the low doses of CBD ameliorated the autism-type social interaction deficits in the mouse model of genetically-induced DS. CBD also increased the GABA inhibitory transmission which was impaired in DS. These therapeutic effects of CBD are mediated through GPR55.	DS	[98]
Adult male C57BL/6 mice	5 mg/kg	5 days a week for 5 weeks	Abnormal-CBD, but not CBD, ameliorated MPTP-induced motor damage. Instead, both compounds significantly reduced the density of microglial cells in the cell body. In the haloperidol-induced catalepsy mouse model, abnormal-CBD also showed anti-cataleptic effects, through the GPR55-activation.	Parkinson’s disease	[99]
Male and female C57BL/6 mice	5–10 and 50 mg/kg	Increasing doses from 5 to 10 mg/kg three times per week, or daily, at a dose of 50 mg/kg, for 23 days	CBD, both at low and high doses, ameliorated the EAE disease. Moreover, CBD treatment reduced the vitality of encephalitogenic cells, levels of IL-6, production of ROS with consequent decrease of the apoptosis process. Additionally, it decreased the levels of GPR55 receptors in the CNS.	EAE disease	[100]

CBD, cannabidiol; DS, Dravet syndrome; GABA, γ-aminobutyric acid; MPTP, 1-methyl-4-phenyl-1,2,3,6-tetrahydropyridine; EAE, experimental autoimmune encephalomyelitis; ROS, reactive oxygen species; CNS, central nervous system.

**Table 4:** Neuroprotective effects of CBD in different neurological diseases through the activation of the TRPV receptors.

In Vitro and in Vivo Models	CBD Dose	Treatments	Biological/Pharmacological Effect	Neurological Diseases	Ref.
Male Wistar rats	10 mg/kg	2 h after the induction of model	CBD inhibited the carrageenan-induced hyperalgesia through the desensitization of the TRPV1 receptor	Hyperalgesia	[31]
hPBMECs and hCMEC/D3 Cells	0.1, 0.3, 1, 3, 10 and 15 μM	7 or 24 h of incubation	CBD, in a dose-dependent manner, led a last-lasting increase in intracellular Ca^2+^ level, through activation of TRPV2. In this way, CBD, enhanced cell proliferation, cell migration and tubulogenesis in human brain endothelial cells.	-	[109]
U87MG glioma cell line	10 µM	Cells were treated with different doses of CBD for 1 day or co-treated with CBD 10 µM and chemotherapeutic drugs for 6 h.	CBD, through activation of TRPV2 and the consequent entry of Ca^2+^, improved the action of chemotherapy drugs enhancing drug absorption and ameliorated cytotoxic activity in human glioma cells.	-	[110]
human Gingival Mesenchymal Stem Cells	5 μM	24 h of incubation	CBD, through TRPV1 desensitization, promoted the PI_3_K/Akt pathway signaling, which can reduce Alzheimer’s hallmarks.	Alzheimer’s disease	[37]

CBD, cannabidiol; hPBMECs, human primary brain microvascular endothelial cell.

**Table 5:** Neuroprotective effects of CBD in different neurological diseases through the activation of the PPARγ.

In Vitro and in Vivo Models	CBD Dose	Treatments	Biological/Pharmacological Effect	Neurological Diseases	Ref.
SH-SY5Y^APP+^	10^−9^–10^−6^ M	24 h	CBD reduced the expression of the APP protein, as well as its ubiquitination, thus leading to the reduction of Aβ and neuronal apoptosis. These CBD’s effects were mediated by PPARγ activation.	Alzheimer’s disease	[64]
Cultures primary of astrocytes rat and male Sprague-Dawley rats	10^−9^–10^−7^ M for in vitro study; 10 mg/kg, for in vivo study.	Daily for 15 days	In the in vitro study, CBD in a concentration-dependent manner reduced the effect of Aβ mediated through the inhibition of NF-κB. In addition, in vivo, CBD ameliorated neuronal damage induced by Aβ and led to a reduction of gliosis and glial fibrillary acidic protein. CBD exerts these effects through PPARγ activation.	Alzheimer’s disease	[17]
Hippocampal slices from C57Bl/6 mice	10 µM	30 min before to the addition of Aβ	The treatment with CBD improved the synaptic transmission and the potentiation long-term in the hippocampus slice of C57/black 6 mice, thereby preserving it from cognitive deficits induced by Aβ_1–42_. CBD exerts these effects, at least in part, through interaction with PPARγ.	Alzheimer’s disease	[117]
Primary microglial cultures from brain of male and female newborn C57/BL6 mice and Swiss mice	60 mg/kg; for in vivo study; 10 µM for in vitro study	Two daily injections 30 min before received haloperidol for 21 days	In mice, CBD treatment prevented dyskinesia induced by haloperidol. Moreover, in the corpus striatum, CBD reduced oxidative stress, activation of microglial, inflammatory cytokine (such as IL-1β and TNF-α) and increased anti-inflammatory cytokine IL-10. It was demonstrated that PPARγ is a molecular target of CBD. In the same way, it was also confirmed the effect of CBD through PPARγ on lipopolysaccharide-stimulated microglial cells.	Tardive dyskinesia	[119]
Male adult C57 ⁄ BL6 mice	15, 30 and 60 mg/kg	15 min before the l-DOPA administration for three days	CBD alone was not able to prevent the l-DOPA-induced dyskinesia. The co-treatment with CBD and capsazepine, through the interaction with CB1 and PPARγ receptors, ameliorate dyskinesia.	Parkinson’s disease	[120]
Human brain microvascular endothelial cell and human astrocyte co-cultures modeled	100 nM, 1 and 10 μM	Either before or immediately after the induction of ischemic damage	CBD (10 μM) prevented the enhance of BBB permeability following the ischemic damage induced by oxygen-glucose deprivation, through the activation of PPARγ and 5-HT_1A_ receptors.	Ischemic stroke	[121]
Male C57BL/6 mice	10 mg/kg	Daily treated, approximately 14 days after disease induction, for 14 days	CBD treatment ameliorated the clinical evidence of disease in EAE mice. CBD restored the PI_3_K/Akt/mTOR pathway that was downregulated after EAE induction. Moreover, CBD reduced inflammatory cytokines IFN-γ and IL-17 significantly and increased the levels of PPARγ. Probably, the anti-inflammatory effects of CBD are linked to the increased of PPARγ.	EAE disease	[122]

CBD, cannabidiol; SH-SY5Y^APP+^, SH-SY5Y cells transfected with the amyloid precursor protein; Aβ, amyloid-β; IL-1β, interleukin 1-β; IL-6, interleukin 6; TNF-α, tumor necrosis factor-α; l-DOPA, l-3,4-dihydroxyphenylalanine; BBB, blood–brain barrier; IFN-γ, interferon-γ; IL-17, interleukin-17; EAE, experimental autoimmune encephalomyelitis.

**Table 6:** Neuroprotective effects of CBD in different neurological diseases through positive allosteric modulation of GABA_A_ receptors.

In Vitro and in Vivo Models	CBD Dose	Treatments	Biological/Pharmacological Effect	Neurological Diseases	Ref.
Surgical human DS and TSC cortical tissue in *Xenopus oocytes*	5 μM	Pre-incubation of cells of 10 s before the co-application of GABA and CBD	CBD, through positive modulation of GABA_A_ receptors, enhanced the amplitude of the GABA-evoked current, in brain tissues of patients with DS and TSC.	DS and TSC	[134]
Male and female *Scn1a^+/−^* mice and *Xenopus oocytes* expressing GABA_A_ receptors	12 mg/kg or 100 mg/kg for in vivo study; 10 µM for in vitro study	In in vivo study, CBD was administrated i.p. 45 min before CLB; in in vitro study CBD (10 µM) was co-applied with GABA, for 60 s	CBD significantly increased the concentrations of CLB and its active metabolite N-CLB, both in the plasma and in the brain. Co-administration of both compounds significantly increased the anticonvulsant effect. CBD and CLB exert their anticonvulsant action by enhancing the activity of the GABA_A_ receptor.	DS	[142]

CBD, cannabidiol; GABA, γ-aminobutyric acid; DS, Dravet syndrome; TSC, tuberous sclerosis complex; *Scn1a^+/−^*, heterozygous loss of function *SCN1A;* CLB, clobazam; *N*-CLB, *N*-desmethylclobazam.

## Abbreviations

CBD	cannabidiol
Δ^9^-THC	Δ^9^-tetrahydro-cannabinol
Nrf2	nuclear factor erythroid 2 – related factor 2
SOD	superoxide dismutase
GSH	glutathione
ROS	reactive oxygen species
iNOS	inducible nitric oxide synthase
IL-6	interleukin-6
IL-1β	interleukin-1β
TNF-α	tumor necrosis factor α
NF-κB	nuclear factor κB
PPARγ	peroxisome proliferator-activated receptor γ
TRP	transient receptor potential
TRPV	transient receptor potential vanilloid
CB1	cannabinoid receptor type 1
CB2	cannabinoid receptor type 2
2-AG	2-arachidonoyl glycerol
AEA	arachidonoylethanolamide
ECS	endocannabinoid system
Ca^2+^	calcium
FAAH	fatty acid amide hydrolase
GABA	γ-aminobutyric acid
5-HT	Serotonin
GPCRs	G protein-coupled receptors
ARs	adenosine receptors
BBB	blood–brain barrier
CYPs	cytochrome P450 enzymes
7-OH-CBD	7-hydroxycannabidiol
G_i/o_	inhibiting G
cAMP	cyclic adenosine monophosphate
PI_3_K/Akt	phosphoinositide 3-kinases/protein kinase B
CNS	central nervous system
TMEV	Theiler’s murine encephalomyelitis virus
i.p.	intraperitoneal
VCAM-1	vascular cell adhesion molecule-1
CCL2	chemokine 2
CCL5	chemokine 5
COX-2	cyclooxygenase-2
Aβ	amyloid-β
BDNF	brain-derived neurotrophic factor
K^+^	potassium
MCA	middle cerebral artery occlusion
PTZ	pentylenetetrazole
i.v.	intravenous
GPR55	G protein-coupled receptors 55
ERK	extracellular receptor-activated kinases
DS	Dravet syndrome
MPTP	1-methyl-4-phenyl-1,2,3,6-tetrahydropyridine
EAE	experimental autoimmune encephalomyelitis
TRPA1	transient receptor potential ankyrin 1
TRPM8	transient receptor potential cation channel subfamily M member 8
hPBMECs	human Primary Brain Microvascular Endothelial Cells
GSK-3β	glycogen synthase kinase 3β
SH-SY5Y^APP+^	SH-SY5Y cells transfected with the amyloid precursor protein
l-DOPA	l-3,4-Dihydroxyphenylalanine
6-OHDA	6-hydroxydopamine
AA-5-HT	arachidonoyl-serotonin
IFN-γ	interferon-γ
IL-17	interleukin-17
TSC	tuberous sclerosis complex
CLB	clobazam
*N*-CLB	*N*-desmethylclobazam
*Scn1a^+/−^*	heterozygous loss of function *SCN1A*
